# 2-Tri­fluoro­methyl-10*H*-benzo[4,5]imidazo[1,2-*a*]pyrimidin-4-one

**DOI:** 10.1107/S160053681302401X

**Published:** 2013-09-12

**Authors:** Chandra, K. B. Puttaraju, K. Shivashankar, E. A. Jithesh Babu, M. Mahendra

**Affiliations:** a Department of Studies in Physics, Manasagangotri, University of Mysore, Mysore 570 006, India; b Department of Chemistry, Central College Campus, Bangalore University, Bangalore 560 001, India

## Abstract

In the mol­ecule of the title compound, C_11_H_6_F_3_N_3_O, the three fused rings of the benzo[4,5]imidazo[1,2-*a*]pyrimidine unit are essentially coplanar, the maximum deviation from the mean plane being 0.096 (2) Å. In the crystal, N—H⋯O hydrogen bonds link the mol­ecules into chains running along the *b*-axis direction.

## Related literature

For the bioactivity of benzo[4,5] imidazo[1,2-*a*]-pyrimidine derivatives, see: Abdel-Hafez (2007 ▶); Nunes *et al.* (2005 ▶); Duval *et al.* (2005 ▶); Palacios *et al.* (2007 ▶); Teimouria & Bazhrang (2006 ▶). For bond-length data, see: Allen *et al.* (1987 ▶).
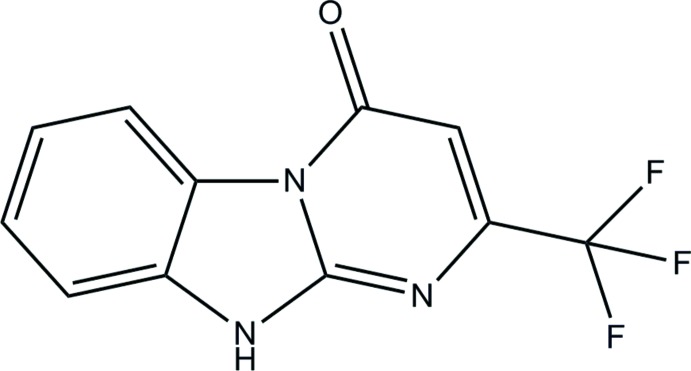


## Experimental

### 

#### Crystal data

C_11_H_6_F_3_N_3_O
*M* *_r_* = 253.19
Monoclinic, 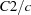

*a* = 20.940 (3) Å
*b* = 13.760 (3) Å
*c* = 7.2852 (11) Å
β = 96.369 (4)°
*V* = 2086.2 (6) Å^3^
*Z* = 8
Mo *K*α radiation
μ = 0.14 mm^−1^
*T* = 273 K
0.30 × 0.25 × 0.20 mm

#### Data collection

Bruker APEXII CCD area-detector diffractometer
9665 measured reflections
1846 independent reflections
1603 reflections with *I* > 2σ(*I*)
*R*_int_ = 0.022

#### Refinement

*R*[*F* ^2^ > 2σ(*F* ^2^)] = 0.050
*wR*(*F* ^2^) = 0.143
*S* = 1.06
1846 reflections
164 parameters
H-atom parameters constrained
Δρ_max_ = 0.42 e Å^−3^
Δρ_min_ = −0.37 e Å^−3^

### 

Data collection: *APEX2* (Bruker, 2009 ▶); cell refinement: *SAINT* (Bruker, 2009 ▶); data reduction: *SAINT*; program(s) used to solve structure: *SHELXS97* (Sheldrick, 2008 ▶); program(s) used to refine structure: *SHELXL97* (Sheldrick, 2008 ▶); molecular graphics: *PLATON* (Spek, 2009 ▶); software used to prepare material for publication: *SHELXL97*.

## Supplementary Material

Crystal structure: contains datablock(s) global, I. DOI: 10.1107/S160053681302401X/ff2117sup1.cif

Structure factors: contains datablock(s) I. DOI: 10.1107/S160053681302401X/ff2117Isup2.hkl

Supplementary material file. DOI: 10.1107/S160053681302401X/ff2117Isup3.cml

Additional supplementary materials:  crystallographic information; 3D view; checkCIF report

## Figures and Tables

**Table 1:** Hydrogen-bond geometry (Å, °)

*D*—H⋯*A*	*D*—H	H⋯*A*	*D*⋯*A*	*D*—H⋯*A*
N1—H1⋯O15^i^	0.86	1.88	2.734 (2)	174

Symmetry code: (i) 
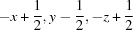
.

**Fig. 1.**
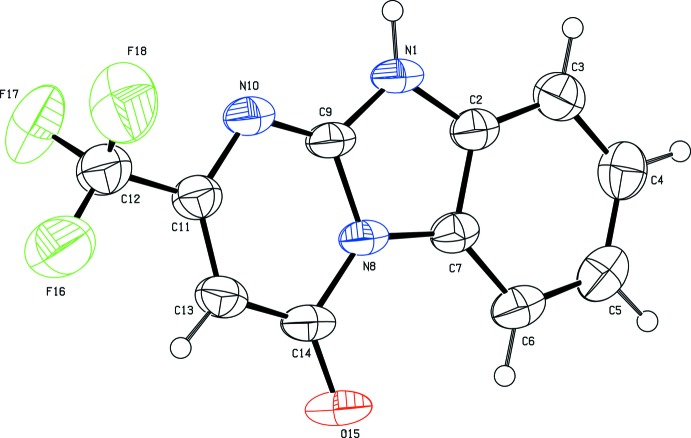
Perspective diagram of the molecule with 50% probability displacement ellipsoids.

**Fig. 2.**
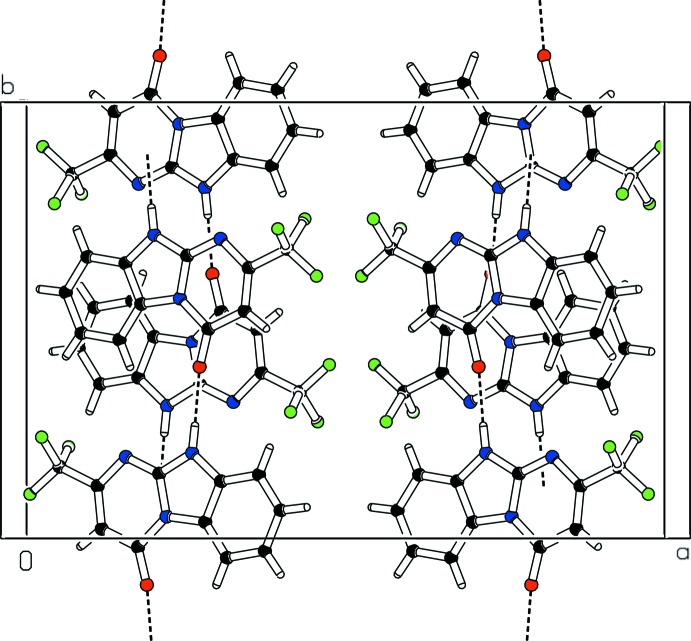
Packing diagram of the molecule viewed down the c axis.

## supplementary crystallographic information

### 1. Comment

Benzo[4,5]imidazo[1,2-*a*]pyrimidin-4-one is a class of fused tricyclic system having three nitrogen atoms. The derivatives of benzopyrimidine are of great importance because of their remarkable biological properties. Some of them have shown good antineoplastic (Abdel-Hafez, 2007) and protein kinase inhibitor (Nunes, Zhu, Amouzegh *et al.*, 2005) activities. Also, heterocycles containing an imidazolone moiety exhibits various biological activities such as antibacterial and antifungal activities (Palacios *et al.*, 2007 and Duval *et al.*, 2005, Teimouria *et al.*, 2006). In view of its extensive background, the title compound was prepared and characterized by single-crystal X-ray diffraction.

In the molecular structure of the title compound (Fig. 1), the bond lengths and angles are generally within normal ranges (Allen *et al.*, 1987). The three fused rings of the benzo[4,5]imidazo[1,2-*a*]pyrimidine unit are essentially coplanar, the maximum deviation from the mean plane being 0.096 (2) Å for atom O15. The crystal structure is stabilized by an intramolecular C—H···O and intermolecular N—H···O hydrogen bonds. The packing diagram of the molecule exhibits linear chain when viewed down the *c* axis as shown in Fig. 2.

### 2. Experimental

An equimolar mixture of 2-aminobenzimadzole (0.5 g, 3.75 mmol) and 4,4,4-Trifluoro-3-oxo-butyric acid ethyl ester (0.69 g, 3.75 mmol) in DMF (10 ml) were added to a microwave tube equipped with a magnetic stir bar. The microwave tube was fitted with a reflux condenser and irradiated in a microwave reactor at a temperature of 130°C for 3 min at a maximum power of 320 W. Then, the reaction mixture was poured on to crushed ice. The solid was filtered and washed with 100 ml of cold water. The crude product was dried and recrystallized from 1:3 ethyl acetate and chloroform to get title compound (Yield = 74%, MP = 223–225°C).

### 3. Refinement

H atoms were placed at idealized positions and allowed to ride on their parent atoms with C–H and N–H distances equal to 0.93 and 0.86 Å, respectively. *U*_iso_(H) = 1.2–1.5*U*_eq_(carrier atom) for all H atoms.

### Crystal data

**Table:**

C_11_H_6_F_3_N_3_O	*F*(000) = 1024
*M**_r_* = 253.19	*D*_x_ = 1.612 Mg m^−^^3^
Monoclinic, *C*2/*c*	Mo *K*α radiation, λ = 0.71073 Å
Hall symbol: -C 2yc	Cell parameters from 1846 reflections
*a* = 20.940 (3) Å	θ = 1.8–25.0°
*b* = 13.760 (3) Å	µ = 0.14 mm^−^^1^
*c* = 7.2852 (11) Å	*T* = 273 K
β = 96.369 (4)°	Block, yellow
*V* = 2086.2 (6) Å^3^	0.30 × 0.25 × 0.20 mm
*Z* = 8	

### Data collection

**Table:**

Bruker APEXII CCD area-detector diffractometer	*R*_int_ = 0.022
ω and φ scans	θ_max_ = 25.0°, θ_min_ = 1.8°
9665 measured reflections	*h* = −24→24
1846 independent reflections	*k* = −16→16
1603 reflections with *I* > 2σ(*I*)	*l* = −8→8

### Refinement

**Table:**

Refinement on *F*^2^	Secondary atom site location: difference Fourier map
Least-squares matrix: full	Hydrogen site location: inferred from neighbouring sites
*R*[*F*^2^ > 2σ(*F*^2^)] = 0.050	H-atom parameters constrained
*wR*(*F*^2^) = 0.143	*w* = 1/[σ^2^(*F*_o_^2^) + (0.0749*P*)^2^ + 1.7218*P*] where *P* = (*F*_o_^2^ + 2*F*_c_^2^)/3
*S* = 1.06	(Δ/σ)_max_ < 0.001
1846 reflections	Δρ_max_ = 0.42 e Å^−^^3^
164 parameters	Δρ_min_ = −0.37 e Å^−^^3^
0 restraints	Extinction correction: *SHELXL97* (Sheldrick, 2008), FC^*^=KFC[1+0.001XFC^2^Λ^3^/SIN(2Θ)]^-1/4^
Primary atom site location: structure-invariant direct methods	Extinction coefficient: 0.0057 (9)

### Special details

**Table:**

Geometry. Bond distances, angles *etc*. have been calculated using the rounded fractional coordinates. All su's are estimated from the variances of the (full) variance-covariance matrix. The cell e.s.d.'s are taken into account in the estimation of distances, angles and torsion angles
Refinement. Refinement on *F*^2^ for ALL reflections except those flagged by the user for potential systematic errors. Weighted *R*-factors *wR* and all goodnesses of fit *S* are based on *F*^2^, conventional *R*-factors *R* are based on *F*, with *F* set to zero for negative *F*^2^. The observed criterion of *F*^2^ > σ(*F*^2^) is used only for calculating -*R*-factor-obs *etc*. and is not relevant to the choice of reflections for refinement. *R*-factors based on *F*^2^ are statistically about twice as large as those based on *F*, and *R*-factors based on ALL data will be even larger.

### Fractional atomic coordinates and isotropic or equivalent isotropic displacement parameters (Å^2^)

**Table:**

	*x*	*y*	*z*	*U*_iso_*/*U*_eq_	
F16	0.47675 (10)	0.39833 (15)	0.5311 (4)	0.1414 (12)	
F17	0.42292 (10)	0.28999 (15)	0.6463 (3)	0.1136 (9)	
F18	0.45363 (9)	0.26760 (18)	0.3881 (3)	0.1191 (9)	
O15	0.29126 (9)	0.60626 (10)	0.2890 (3)	0.0714 (6)	
N1	0.21914 (9)	0.30385 (11)	0.1890 (2)	0.0536 (6)	
N8	0.25963 (8)	0.44972 (11)	0.2391 (2)	0.0447 (5)	
N10	0.32492 (9)	0.31321 (12)	0.3422 (3)	0.0542 (6)	
C2	0.17285 (10)	0.36901 (14)	0.1150 (3)	0.0487 (6)	
C3	0.11203 (12)	0.35408 (18)	0.0256 (3)	0.0600 (8)	
C4	0.07724 (12)	0.43589 (19)	−0.0316 (3)	0.0642 (8)	
C5	0.10216 (12)	0.52902 (18)	−0.0013 (3)	0.0630 (8)	
C6	0.16278 (11)	0.54443 (15)	0.0884 (3)	0.0543 (7)	
C7	0.19775 (10)	0.46220 (13)	0.1455 (3)	0.0456 (6)	
C9	0.27155 (10)	0.35158 (13)	0.2628 (3)	0.0469 (6)	
C11	0.36977 (11)	0.37977 (15)	0.4020 (3)	0.0539 (7)	
C12	0.43075 (12)	0.33566 (19)	0.4922 (4)	0.0703 (9)	
C13	0.36306 (11)	0.47831 (15)	0.3890 (3)	0.0559 (7)	
C14	0.30517 (11)	0.51983 (13)	0.3069 (3)	0.0508 (7)	
H1	0.21480	0.24170	0.18780	0.0640*	
H3	0.09530	0.29190	0.00490	0.0720*	
H4	0.03600	0.42860	−0.09210	0.0770*	
H5	0.07720	0.58230	−0.04260	0.0760*	
H6	0.17940	0.60660	0.10930	0.0650*	
H13	0.39690	0.51840	0.43460	0.0670*	

### Atomic displacement parameters (Å^2^)

**Table:**

	*U*^11^	*U*^22^	*U*^33^	*U*^12^	*U*^13^	*U*^23^
F16	0.0922 (14)	0.0935 (14)	0.221 (3)	−0.0293 (11)	−0.0608 (16)	0.0449 (15)
F17	0.1047 (14)	0.1321 (17)	0.1016 (13)	0.0193 (12)	0.0007 (10)	0.0543 (12)
F18	0.0868 (12)	0.1397 (18)	0.1283 (16)	0.0457 (12)	0.0013 (11)	−0.0196 (14)
O15	0.0854 (12)	0.0276 (7)	0.1030 (13)	−0.0046 (7)	0.0190 (10)	−0.0015 (7)
N1	0.0663 (11)	0.0265 (8)	0.0674 (11)	−0.0028 (7)	0.0048 (9)	−0.0015 (7)
N8	0.0584 (10)	0.0277 (8)	0.0497 (9)	−0.0007 (7)	0.0139 (7)	−0.0005 (6)
N10	0.0630 (11)	0.0337 (9)	0.0653 (11)	0.0000 (8)	0.0046 (9)	0.0000 (7)
C2	0.0595 (12)	0.0390 (10)	0.0489 (11)	0.0001 (8)	0.0118 (9)	0.0002 (8)
C3	0.0668 (14)	0.0560 (13)	0.0573 (12)	−0.0062 (11)	0.0079 (11)	−0.0034 (10)
C4	0.0621 (14)	0.0758 (16)	0.0549 (13)	0.0070 (12)	0.0071 (10)	0.0027 (11)
C5	0.0699 (15)	0.0645 (15)	0.0564 (13)	0.0181 (11)	0.0154 (11)	0.0111 (10)
C6	0.0710 (14)	0.0396 (10)	0.0554 (12)	0.0065 (9)	0.0208 (10)	0.0047 (9)
C7	0.0576 (12)	0.0371 (10)	0.0446 (10)	0.0020 (8)	0.0162 (9)	0.0010 (8)
C9	0.0609 (12)	0.0282 (9)	0.0527 (11)	−0.0008 (8)	0.0106 (9)	−0.0004 (8)
C11	0.0632 (13)	0.0455 (11)	0.0536 (12)	−0.0033 (9)	0.0089 (10)	0.0026 (9)
C12	0.0673 (15)	0.0603 (14)	0.0817 (17)	−0.0031 (12)	0.0017 (13)	0.0072 (13)
C13	0.0650 (13)	0.0440 (11)	0.0592 (12)	−0.0128 (10)	0.0093 (10)	−0.0030 (9)
C14	0.0674 (13)	0.0311 (10)	0.0565 (11)	−0.0066 (9)	0.0189 (10)	−0.0023 (8)

### Geometric parameters (Å, º)

**Table:**

F16—C12	1.301 (3)	C2—C7	1.393 (3)
F17—C12	1.313 (4)	C3—C4	1.380 (4)
F18—C12	1.328 (4)	C4—C5	1.392 (4)
O15—C14	1.228 (2)	C5—C6	1.378 (3)
N1—C2	1.385 (3)	C6—C7	1.386 (3)
N1—C9	1.339 (3)	C11—C13	1.365 (3)
N8—C7	1.406 (3)	C11—C12	1.498 (3)
N8—C9	1.381 (2)	C13—C14	1.412 (3)
N8—C14	1.407 (3)	C3—H3	0.9300
N10—C9	1.311 (3)	C4—H4	0.9300
N10—C11	1.349 (3)	C5—H5	0.9300
N1—H1	0.8600	C6—H6	0.9300
C2—C3	1.380 (3)	C13—H13	0.9300
			
F16···F16^i^	3.013 (3)	C5···C4^vi^	3.551 (3)
F17···N10	2.866 (3)	C5···C7^vii^	3.433 (3)
F17···C3^ii^	3.251 (3)	C6···C4^vi^	3.471 (3)
F18···F18^iii^	2.947 (3)	C6···O15	3.037 (3)
F18···N10	2.751 (3)	C6···N8^vii^	3.426 (3)
F16···H13^i^	2.8700	C6···C5^vi^	3.523 (3)
F16···H13	2.4000	C6···C7^vii^	3.388 (3)
F17···H3^ii^	2.8400	C7···C5^vi^	3.433 (3)
O15···C6	3.037 (3)	C7···C6^vi^	3.388 (3)
O15···N1^iv^	2.734 (2)	C7···C14^vii^	3.527 (3)
O15···H6	2.5500	C13···C14^vi^	3.400 (3)
O15···H1^iv^	1.8800	C14···N8^vi^	3.415 (3)
N1···N8	2.194 (2)	C14···C13^vii^	3.400 (3)
N1···O15^v^	2.734 (2)	C14···C7^vi^	3.527 (3)
N8···N1	2.194 (2)	C5···H4^viii^	3.1000
N8···C6^vi^	3.426 (3)	C14···H6	3.1000
N8···C14^vii^	3.415 (3)	C14···H1^iv^	3.0800
N10···F17	2.866 (3)	H1···O15^v^	1.8800
N10···F18	2.751 (3)	H1···C14^v^	3.0800
N10···H6^v^	2.8700	H3···F17^ii^	2.8400
C2···C5^vi^	3.591 (3)	H4···C5^viii^	3.1000
C3···F17^ii^	3.251 (3)	H6···O15	2.5500
C4···C5^vii^	3.551 (3)	H6···C14	3.1000
C4···C6^vii^	3.471 (3)	H6···N10^iv^	2.8700
C5···C6^vii^	3.523 (3)	H13···F16	2.4000
C5···C2^vii^	3.591 (3)	H13···F16^i^	2.8700
			
C2—N1—C9	110.23 (16)	N10—C11—C12	113.30 (19)
C7—N8—C9	108.92 (16)	F16—C12—C11	113.7 (2)
C7—N8—C14	129.70 (16)	F16—C12—F17	106.9 (3)
C9—N8—C14	121.38 (17)	F16—C12—F18	106.7 (2)
C9—N10—C11	113.45 (17)	F18—C12—C11	112.3 (2)
C2—N1—H1	125.00	F17—C12—F18	103.8 (2)
C9—N1—H1	125.00	F17—C12—C11	112.8 (2)
N1—C2—C3	131.05 (19)	C11—C13—C14	120.6 (2)
N1—C2—C7	107.49 (18)	N8—C14—C13	112.83 (16)
C3—C2—C7	121.5 (2)	O15—C14—N8	118.9 (2)
C2—C3—C4	116.7 (2)	O15—C14—C13	128.3 (2)
C3—C4—C5	121.8 (2)	C2—C3—H3	122.00
C4—C5—C6	121.8 (2)	C4—C3—H3	122.00
C5—C6—C7	116.4 (2)	C3—C4—H4	119.00
N8—C7—C2	105.87 (16)	C5—C4—H4	119.00
N8—C7—C6	132.28 (18)	C4—C5—H5	119.00
C2—C7—C6	121.9 (2)	C6—C5—H5	119.00
N1—C9—N8	107.50 (17)	C5—C6—H6	122.00
N8—C9—N10	125.63 (18)	C7—C6—H6	122.00
N1—C9—N10	126.87 (17)	C11—C13—H13	120.00
N10—C11—C13	126.1 (2)	C14—C13—H13	120.00
C12—C11—C13	120.6 (2)		
			
C9—N1—C2—C3	179.8 (2)	C7—C2—C3—C4	−0.1 (3)
C9—N1—C2—C7	−0.4 (2)	N1—C2—C7—N8	0.0 (2)
C2—N1—C9—N8	0.6 (2)	N1—C2—C7—C6	−179.6 (2)
C2—N1—C9—N10	−178.9 (2)	C3—C2—C7—N8	179.80 (19)
C9—N8—C7—C2	0.4 (2)	C3—C2—C7—C6	0.2 (3)
C9—N8—C7—C6	179.9 (2)	C2—C3—C4—C5	0.2 (3)
C14—N8—C7—C2	−178.33 (19)	C3—C4—C5—C6	−0.4 (4)
C14—N8—C7—C6	1.2 (4)	C4—C5—C6—C7	0.5 (3)
C7—N8—C9—N1	−0.6 (2)	C5—C6—C7—N8	−179.9 (2)
C7—N8—C9—N10	178.9 (2)	C5—C6—C7—C2	−0.4 (3)
C14—N8—C9—N1	178.23 (17)	N10—C11—C12—F16	172.5 (2)
C14—N8—C9—N10	−2.2 (3)	N10—C11—C12—F17	−65.5 (3)
C7—N8—C14—O15	1.5 (3)	N10—C11—C12—F18	51.3 (3)
C7—N8—C14—C13	−178.26 (19)	C13—C11—C12—F16	−8.5 (4)
C9—N8—C14—O15	−177.0 (2)	C13—C11—C12—F17	113.5 (3)
C9—N8—C14—C13	3.2 (3)	C13—C11—C12—F18	−129.7 (2)
C11—N10—C9—N1	179.3 (2)	N10—C11—C13—C14	−0.1 (4)
C11—N10—C9—N8	−0.1 (3)	C12—C11—C13—C14	−178.9 (2)
C9—N10—C11—C12	−179.9 (2)	C11—C13—C14—O15	178.1 (2)
C9—N10—C11—C13	1.3 (3)	C11—C13—C14—N8	−2.1 (3)
N1—C2—C3—C4	179.7 (2)		

Symmetry codes: (i) −*x*+1, −*y*+1, −*z*+1; (ii) −*x*+1/2, −*y*+1/2, −*z*+1; (iii) −*x*+1, *y*, −*z*+1/2; (iv) −*x*+1/2, *y*+1/2, −*z*+1/2; (v) −*x*+1/2, *y*−1/2, −*z*+1/2; (vi) *x*, −*y*+1, *z*+1/2; (vii) *x*, −*y*+1, *z*−1/2; (viii) −*x*, −*y*+1, −*z*.

### Hydrogen-bond geometry (Å, º)

**Table:**

*D*—H···*A*	*D*—H	H···*A*	*D*···*A*	*D*—H···*A*
N1—H1···O15^v^	0.86	1.88	2.734 (2)	174
C6—H6···O15	0.93	2.55	3.037 (3)	113
C13—H13···F16	0.93	2.40	2.721 (3)	100

Symmetry code: (v) −*x*+1/2, *y*−1/2, −*z*+1/2.
